# Multiparametric [^18^F]Fluorodeoxyglucose/ [^18^F]Fluoromisonidazole Positron Emission Tomography/ Magnetic Resonance Imaging of Locally Advanced Cervical Cancer for the Non-Invasive Detection of Tumor Heterogeneity: A Pilot Study

**DOI:** 10.1371/journal.pone.0155333

**Published:** 2016-05-11

**Authors:** Katja Pinker, Piotr Andrzejewski, Pascal Baltzer, Stephan H. Polanec, Alina Sturdza, Dietmar Georg, Thomas H. Helbich, Georgios Karanikas, Christoph Grimm, Stephan Polterauer, Richard Poetter, Wolfgang Wadsak, Markus Mitterhauser, Petra Georg

**Affiliations:** 1 Department of Biomedical Imaging and Image-guided Therapy, Division of Molecular and Gender Imaging, Medical University of Vienna, Vienna, Austria; 2 Christian Doppler Laboratory for Medical Radiation Research for Radiation Oncology, Medical University of Vienna,Vienna, Austria; 3 Department of Radiation Oncology, Medical University of Vienna, Vienna, Austria; 4 Department of Biomedical Imaging and Image-guided Therapy, Division of Nuclear Medicine, Medical University of Vienna, Vienna, Austria; 5 Department of Gynecology and Obstetrics, Medical University of Vienna, Vienna, Austria; 6 Gynecologic Cancer Unit/Comprehensive Cancer Center Vienna, Medical University of Vienna, Vienna, Austria; 7 Department of Scientific Computing in Medicine, State University of Florida, Tallahassee, Florida, United States of America; University of Graz, AUSTRIA

## Abstract

**Objectives:**

To investigate fused multiparametric positron emission tomography/magnetic resonance imaging (MP PET/MRI) at 3T in patients with locally advanced cervical cancer, using high-resolution T2-weighted, contrast-enhanced MRI (CE-MRI), diffusion-weighted imaging (DWI), and the radiotracers [^18^F]fluorodeoxyglucose ([^18^F]FDG) and [^18^F]fluoromisonidazol ([^18^F]FMISO) for the non-invasive detection of tumor heterogeneity for an improved planning of chemo-radiation therapy (CRT).

**Materials and Methods:**

Sixteen patients with locally advanced cervix were enrolled in this IRB approved and were examined with fused MP [^18^F]FDG/ [^18^F]FMISO PET/MRI and in eleven patients complete data sets were acquired. MP PET/MRI was assessed for tumor volume, enhancement (EH)-kinetics, diffusivity, and [^18^F]FDG/ [^18^F]FMISO-avidity. Descriptive statistics and voxel-by-voxel analysis of MRI and PET parameters were performed. Correlations were assessed using multiple correlation analysis.

**Results:**

All tumors displayed imaging parameters concordant with cervix cancer, i.e. type II/III EH-kinetics, restricted diffusivity (median ADC 0.80x10^-3^mm^2^/sec), [^18^F]FDG- (median SUV_max_16.2) and [^18^F]FMISO-avidity (median SUV_max_3.1). In all patients, [^18^F]FMISO PET identified the hypoxic tumor subvolume, which was independent of tumor volume. A voxel-by-voxel analysis revealed only weak correlations between the MRI and PET parameters (0.05–0.22), indicating that each individual parameter yields independent information and the presence of tumor heterogeneity.

**Conclusion:**

MP [^18^F]FDG/ [^18^F]FMISO PET/MRI in patients with cervical cancer facilitates the acquisition of independent predictive and prognostic imaging parameters. MP [^18^F]FDG/ [^18^F]FMISO PET/MRI enables insights into tumor biology on multiple levels and provides information on tumor heterogeneity, which has the potential to improve the planning of CRT.

## Introduction

Chemo-radiation therapy (CRT) is the standard of care for locally advanced cervical cancer and improves local control and survival [1]. The role of advanced imaging is steadily increasing in the management of gynecological malignancies, both for treatment planning and response monitoring [2–10]. Magnetic resonance imaging (MRI) and positron emission tomography (PET) play a pivotal role in planning and monitoring the response to CRT [2–6, 11–14]. MRI provides morphological and functional information on tumor neo-angiogenesis, perfusion, and tissue cellularity, using multiple parameters, such as T2-weighted, contrast-enhanced (CE), and diffusion-weighted imaging (DWI) [15–22]. PET, using the radiotracer 2-deoxy-2-[^18^F]fluoro-D-glucose ([^18^F]FDG), provides metabolic information by depicting glycolytic tumor activity [13, 14].

In locally advanced cervical cancer, it is known for a long time that tumor hypoxia is associated with increased resistance to CRT, thus diminishing the rate of local control as well as distant disease control [23–30]. Traditional clinical methods to determine hypoxic regions are invasive and based on needle electrodes or tissue sampling [23, 27, 28, 31]. PET imaging using [^18^F]fluoromisonidazole (1-[^18^F]fluoro-3-(2-nitroimidazol-1-yl)propan-2-ol or short [^18^F]FMISO) can identify hypoxic tumor sub-volumes and track spatio-temporal dynamics. Therefore it might be of considerable additional value for improved planning and monitoring of CRT for cervix cancer [23–28, 32, 33].

To date, the potential of PET/MRI for locally advanced cervical cancer, using multiple MRI parameters and different radiotracers in the assessment of cervical cancer, has not been explored.

We hypothesized that through the non-invasive quantitative assessment of multiple processes relevant for cancer growth, progression and aggressiveness (tumor neo-angiogenesis and perfusion, cellularity, glycolytic metabolic activity, tumor hypoxia) [34] hitherto unparalleled insights into tumor biology on multiple levels could be provided by multiparametric (MP) [^18^F]FDG/ [^18^F]FMISO PET/MRI, which can be subsequently used for treatment stratification and intensification.

The aim of this study was to explore MP MRI and PET in patients with locally advanced cervix cancer. Thus, the aim of our study was to assess whether fused MP [^18^F]FDG/ [^18^F]FMISO PET/MRI in cervical cancer patients is possible and facilitates information on tumor heterogeneity, which might improve the planning of CRT. To reach this goal, we used multiple MRI parameters (high-resolution T2-weighted-, CE-MRI, DWI) and the combination of these parameters with the radiotracers [^18^F]FDG, for the assessment of glycolytic metabolic activity, and [^18^F]FMISO, for the detection of tumor hypoxia with PET.

## Materials and Methods

### Patients

From 05/2012 to 07/2014, sixteen consecutive patients (mean age, 51.8; range, 36–72), who presented at the Department of Radiation Oncology for treatment, were included in this prospective, single-institution feasibility study, which was approved by the institutional review board (IRB)/ Ethics Committee of the Medical University of Vienna, Austria. All patients fulfilled the following inclusion criteria and underwent dual tracer MP PET/MRI: 18 years or older; histopathologically verified locally advanced cervical cancer scheduled for treatment with CRT; not pregnant; not breastfeeding; no previous treatment; and no contraindications for MRI or contrast agents. Written, informed consent was obtained from all patients. Examinations of 11 patients were completed within a median of one week (range 2–23) and were used for this study. The remaining patients did either not want to complete the study or treatment could not be delayed for the imaging study.

### Imaging

Prior to commencement of treatment all patients underwent fused PET/MRI with PET/computed tomography (CT), using [^18^F]FDG and [^18^F]FMISO and MP MRI of the pelvis at 3T.

#### PET/CT

A hybrid PET-CT (computed tomography) (Biograph 64 TruePoint PET/CT system, Siemens, Erlangen/Germany) was used. For [^18^F]FDG PET/CT, patients fasted for five hours and blood glucose levels were <150 mg/dl (8.3mmol/l). All patients received a body-weight-adapted injection of approximately 200-350MBq [^18^F]FDG and [18F]FMISO on different days. Scanning was started after an uptake time of 60 min for [^18^F]FDG and 210-240min for [^18^F]FMISO. For both radiotracers, a supine PET dataset of unenhanced CT scans were recorded for attenuation correction. For [^18^F]FMISO, the unenhanced CT scans were obtained as low-dose scans. The same imaging and post-recon parameters were used for both PET/CT studies. PET images were reconstructed using the iterative TrueX algorithm, which incorporates a specific correction for the point-spread function in addition to commonly used correction factors [35, 36]. Four iterations per 21 subsets were used, with a matrix size of 168×168, a trans-axial field of view of 605 mm (pixel size 3.6mm), and a section thickness of 5mm. Further technical details are provided by the manufacturer [37].

#### MP MR Imaging

All MRI studies were performed with the patient in the supine position using a 3T MRI (Tim-Trio, Siemens, Erlangen/Germany). A combination of an eight-channel spine array (24 elements in eight clusters) and a two-channel body array (six elements in two clusters) was used for signal acquisition. The MRI protocol consisted of:

1. A sagittal T2-weighted turbo spin echo (TSE) sequence: time to repetition (TR)/ echo time (TE) 4630/89msec; field of view (FOV) 220mm; 30 slices; voxel size 0.7 x 0.6 x 3mm^3^, three averages; acquisition time (TA) 5:16min).
2. A sagittal 3D slab-selective T2-weighted TSE sequence (Sampling Perfection with Application optimized Contrasts using different flip angle Evolution, SPACE): TR/TE 1500/173ms; FOV 300mm; 176 slices per slab; 0.9mm^3^ isotropic; two averages; TA 3:56min.
3. An axial T1-weighted TSE sequence: TR/TE 675/12; FOV 280mm; 30 slices per slab; voxel size 0.6 x 0.6 x 3mm^3^; 2 averages; TA 4:19min.
4. An axial diffusion-weighted 2D echo-planar imaging sequence with spectrally adiabatic inversion recovery (SPAIR) fat suppression (TR/TE 6300/82ms; FOV 28mm; 30 slices voxel size 1.8 x 1.5 x 5mm^3^; five averages; b-values 50 and 850sec/mm^2^ ([38, 39]; TA 3:28min.
5. An axial T1-weighted Volume Interpolated Breath-hold Examination (VIBE) sequence with SPAIR fat suppression before, 1min and 4min after contrast agent application (TR/TE 3.38/1.38ms; FOV 380mm; 52 slices per slab; voxel size 0.8 x 0.8 x 3mm^3^; one average; TA 0:53min).

Gadoteratemeglumine (Gd-DOTA;Dotarem^®^, Guerbet, France) was injected intravenously as a bolus (0.1mmol/kg body weight) using a power injector at 4 ml/s, followed by a 20mL saline flush. The total MRI examination time was ~16min.

### Image Fusion

#### Rigid registrations for descriptive statistics

**Fig 1:**
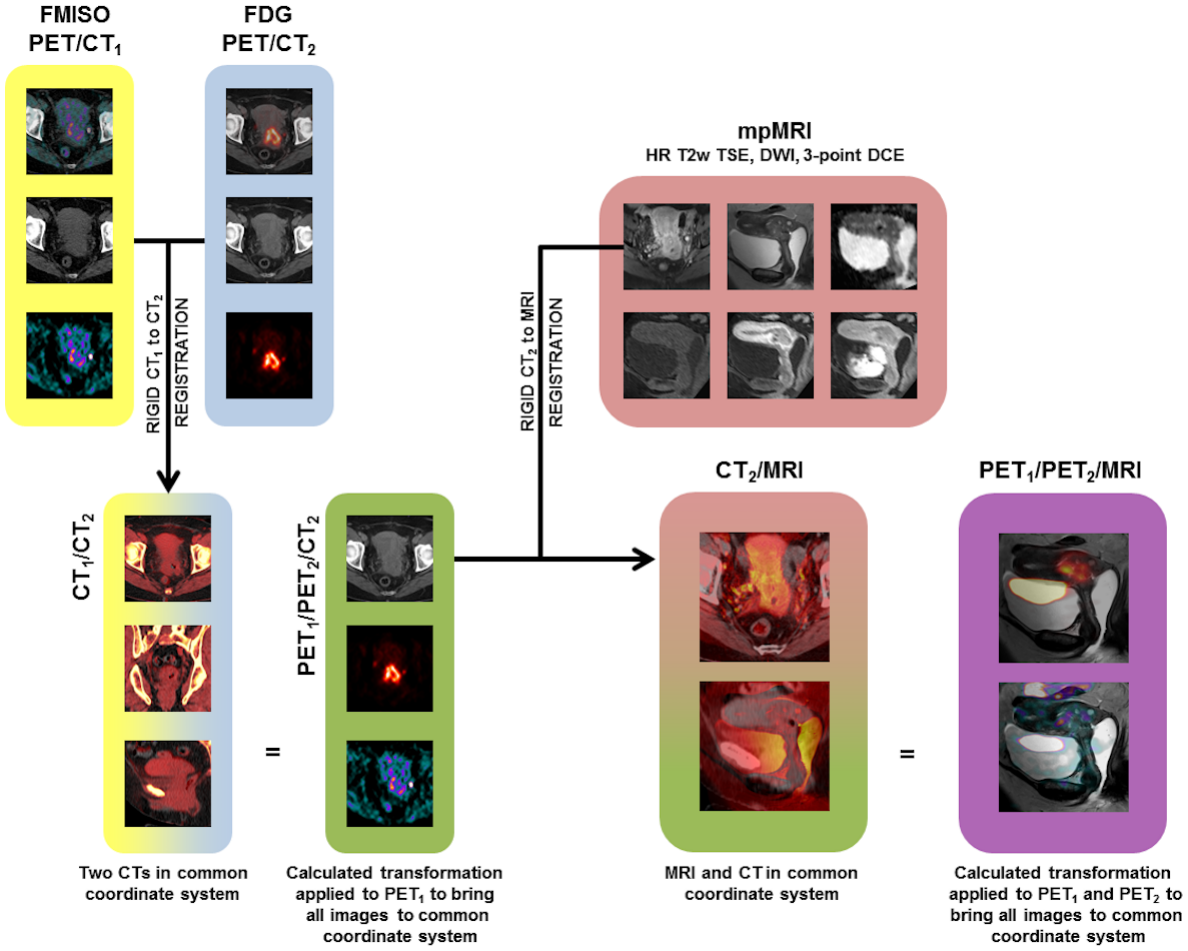
Step-by-step illustration of the rigid registration algorithm. [^18^F]FMISO and [^18^F]FDG/ PET/CT datasets in two separate coordinate systems (yellow and blue) are rigidly registered (CT_1_-CT_2_ registration) and merged into one (green). Registration between the green and the red (mpMRI) coordinate system is performed by a rigid transformation of CT_2_ to fit T1w MRI. The calculated transformation is applied to PET_1_ and PET_2_ to create the PET/MRI dataset (purple).

For descriptive statistics, the images were analyzed and registered using Mirada RTx software (Mirada Medical Ltd., UK). Datasets acquired on the same scanners, i.e. PET/CT and MP MRI data, were initially fused according to DICOM tag information. The correlation of the anatomy on respective modalities was visually checked by the readers in consensus and adjusted if necessary utilizing the available software options (automatic, mutual information-based, and manual rigid registration). To achieve optimal registration fusion of [^18^F]FDG, [^18^F]FMISO, and MP MRI the registration was performed in two steps (visualized as arrows in Fig 1). First, the CT series of the respective PET-CT examinations were registered and the two PET series were saved in the same coordinate system. Subsequently, the registration between PET and MP MRI datasets was performed using CT and anatomic T2-weighted MR images. In both steps, the automatic and manual rigid registration tools were used with a special focus on the cervix.

#### Deformable image registrations for voxel-by-voxel analysis

For voxel-by-voxel analysis deformable image registration (DIR) was applied. In this step the uterus, cervix as well as the different helper structures were defined on CT and projected onto [^18^F]FDG/ [^18^F]FMISO PET/CT and axial T1-weighted MRI data. All images were resampled to the voxel size of 0.6 x 0.6 x 3mm^3^. Hybrid-, intensity-, and structure-based DIR were performed for MP MRI and [^18^F]FDG, as well as for [^18^F]FMISO CT data using RayStation (ver X4.6.100, RaySearch Laboratories, Sweden) (see S1 Fig). The uterus was used as the controlling structure and helper structures were used as focus structures to limit the registration calculations to this region.

### Data analysis

An experienced radiologist and an experienced nuclear medicine physician in consensus prospectively evaluated MP [^18^F]FDG/ [^18^F]FMISO PET/MRI data according to the criteria listed below:

#### High-resolution T2-weighted and CE-MRI

CE-MRI imaging data was assessed for tumor morphology, and for initial and delayed enhancement kinetics. For analysis of lesion enhancement kinetics in the early (1min) and delayed phase (5min), manually drawn region-of interests (ROI) were placed in the most enhancing parts of a lesion and the intensity courses were plotted against time. Initial enhancement (IE) was defined as either medium (<1.5) or fast (>1.5), and enhancement in the delayed phase was defined as either wash-out (<-0.1), plateau (>-0.1 and <0.1), or persistent (>0.1). The lower the rate the faster the wash-out (WO). The definitions of IE and WO are as follows;

$$\text{IE}=\frac{{\text{I}}_{{\text{DCE}}_{\text{early }}}+{\text{I}}_{{\text{DCE}}_{\text{native}}}}{{\text{I}}_{{\text{DCE}}_{\text{native}}}}$$

$$\text{WO}=\frac{{\text{I}}_{{\text{DCE}}_{\text{late }}}+{\text{I}}_{{\text{DCE}}_{\text{early}}}}{{\text{I}}_{{\text{DCE}}_{\text{native}}}}$$

Lesion volume in cc was calculated by measuring the largest diameter of the tumor in all three planes on the CE-MRI.

#### DWI

High-b-value DW images (i.e., 850s/mm^2^) were visually assessed for hyperintense areas that corresponded to the morphologically visible tumor on T2-weighted and CE-MRI. Two-dimensional ROIs were manually drawn covering the area, visually assessed, with the lowest ADC values inside the lesion, and the mean ADC was recorded. Partial volume effects due to normal parenchyma, suppressed fatty tissue, and areas of necrotic tissue, as identified from the morphological and contrast-enhanced images, were avoided as far as possible.

#### [^18^F]FDG and [^18^F]FMISO PET

Tumor uptake was quantified by maximum standardized uptake values (SUV_MAX_). For SUV_MAX_ determination, the reader placed a sphere around the lesion. This sphere encompassed the entire lesion, but excluded physiologic [^18^F]FDG uptake in surrounding tissues. In addition, tumor-to-background ratio was calculated using the gluteal muscle for [^18^F]FMISO and mediastinal up-take for [^18^F]FDG as background.

### Voxel-by-voxel analysis

**Fig 2:**
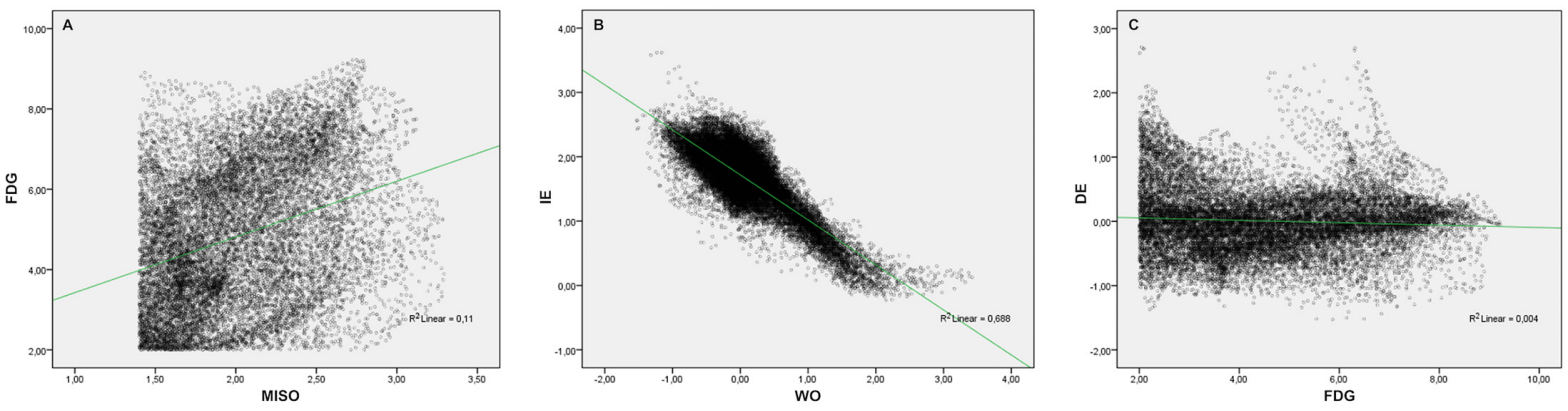
Representative examples of voxel-by-voxel correlations between pairs of PET/MRI dataset-derived parameters. (A) illustrates a weak correlation of the evaluated parameters, i.e., [^18^F]FMISO- and [^18^F]FDG-avidity. (B) shows a strong indirect correlation of the evaluated parameters, i.e., initial and delayed enhancement. (C), there is no correlation of the evaluated parameters, i.e., delayed enhancement and [^18^F]FDG-avidity.

The voxel-by-voxel analysis of all MRI and PET parameters on the delineated cervix was performed using an in-house-developed MATLAB script. Due to the lower image resolution of PET images and ADC maps, and to compensate for potential inaccuracies of registration between the imaging modalities, a slice-wise sliding window of 24 pixels surrounding the pixel of interest (middle pixel) was used to calculate the average of the measured parameters in this area. Values between the 0.5^th^ and 99.5^th^ percentiles were taken into consideration. Tumor-to-background ratio maps for PET, as well as initial and delayed enhancement maps for CE-MRI, were calculated. Fig 2 illustrates representative voxel-by-voxel correlations between pairs of PET/MRI dataset-derived parameters.

#### Statistical analysis

Statistical analyses were performed using Statistical Package for the Social Sciences (IBM SPSS Statistics 22.0). Correlations between items were investigated by constructing a color-coded nonparametric Spearman’s rank correlation coefficient matrix. P-values <0.05 were considered significant (a two-tailed significance test was used). Correlations of voxel-by-voxel analysis between the imaging parameters were assessed with the nonparametric Spearman’s rank correlation coefficient and by plotting a set of 10 scatter plots for each patient, which combined each pair of investigated parameters. In PET datasets, only voxels with a tumor-to-background ratio higher than 2, for [^18^F]FDG, and/or 1.4, for [^18^F]FMISO, were taken into account. For all parameter pairs, the average correlation coefficients that resulted from the patient-wise analysis were calculated using Fisher Z-transformation, and a color-coded correlation coefficient matrix was plotted.

## Results

Image registration between the various MRI sequences and PET image series was successfully performed in all eleven patients with complete data sets. Table 1 summarizes the various qualitative (e.g. WO, IE) and quantitative imaging parameters (e.g. ADC, SUV) extracted.

**Table 1:** Patients’ age, histopathological diagnoses, tumor volumes, qualitative, i.e WO, IE and quantitative imaging parameters, i.e ADC, [^18^F]FDG and [^18^F]FMISO SUVmax and [^18^F]FMISO TBR for all patients with complete data sets.

ID	Age	Histopathology	Tumor Volume (cm^3^)	IE	WO	ADC (x 10^−3^ mm^2^/sec)	SUV_max_ [^18^F]FDG	SUV_max_ [^18^F]FMISO	[^18^F]FMISO TBR
1	36	SCC	128.7	Fast	Wash-out	0.82	16.1	2.8	2.5
2	58	SCC	440	Fast	Wash-out	0.77	15	3.1	2.8
3	56	SCC	277.4	Medium	Wash-out	0.53	25.6	6	4.6
4	36	SCC	108.4	Medium	Wash-out	0.67	11.9	2.4	2.0
5	38	SCC	111.3	Medium	Wash-out	0.80	21.5	2.8	2.0
6	72	SCC	87.4	Medium	Wash-out	0.66	16.2	2.9	2.4
7	58	SCC	38	Fast	Plateau	0.89	12.2	4.2	2.8
8	54	SCC	32.1	Fast	Plateau	0.84	19.2	4.6	3.5
9	60	SCC	6.2	Fast	Wash-out	0.85	23.3	3.4	2.6
10	36	SCC	25.3	Medium	Persistent	0.71	12	2.2	2.0
11	66	SCC	52.1	Medium	Persistent	0.91	21.4	6.4	3.8

Note: ID—patient identification number, SCC- squamous cell carcinoma, IE—initial enhancement rate, WO—wash-out rate, ADC—apparent diffusion coefficient, SUV_max_—maximum standard up-take value, [^18^F]FDG—[^18^F]Fluorodeoxyglucose, [^18^F]FMISO—[^18^F]Fluoromisonidazole, TBR—tumor-to-background ratio.

Tumor volumes ranged from 6.2 to 440.0cm^3^ (mean 118.8±124.1cm^3^, median 87.4cm^3^). There was a fast initial enhancement (IE) in five and a medium IE in six patients, followed by either a wash-out (n = 6), a plateau (n = 3), or persistent (n = 2) enhancement. All tumors demonstrated restricted diffusivity, with median ADC values of 0.80 x 10^-3^mm^2^/sec (mean 0.77, range 0.53–0.91, SD 0.11 mm^2^/sec). All tumors were highly [^18^F]FDG-avid with a median SUV_max_ of 16.2 (mean 17.7, range 11.9–25.6, SD 4.6). In all patients [^18^F]FMISO-avid spots were identified within the [^18^F]FDG-avid lesion (see S2 Fig). With [^18^F]FMISO there was a median SUV_max_ of 3.1 (mean 3.7, range 2.2–6.4, SD 1.4). Median [^18^F]FMISO tumor-to-background ratio was 2.6 (mean 2.8, range 2.0–4.6, SD 0.8).

### Lesion-based descriptive statistics

**Fig 3:**
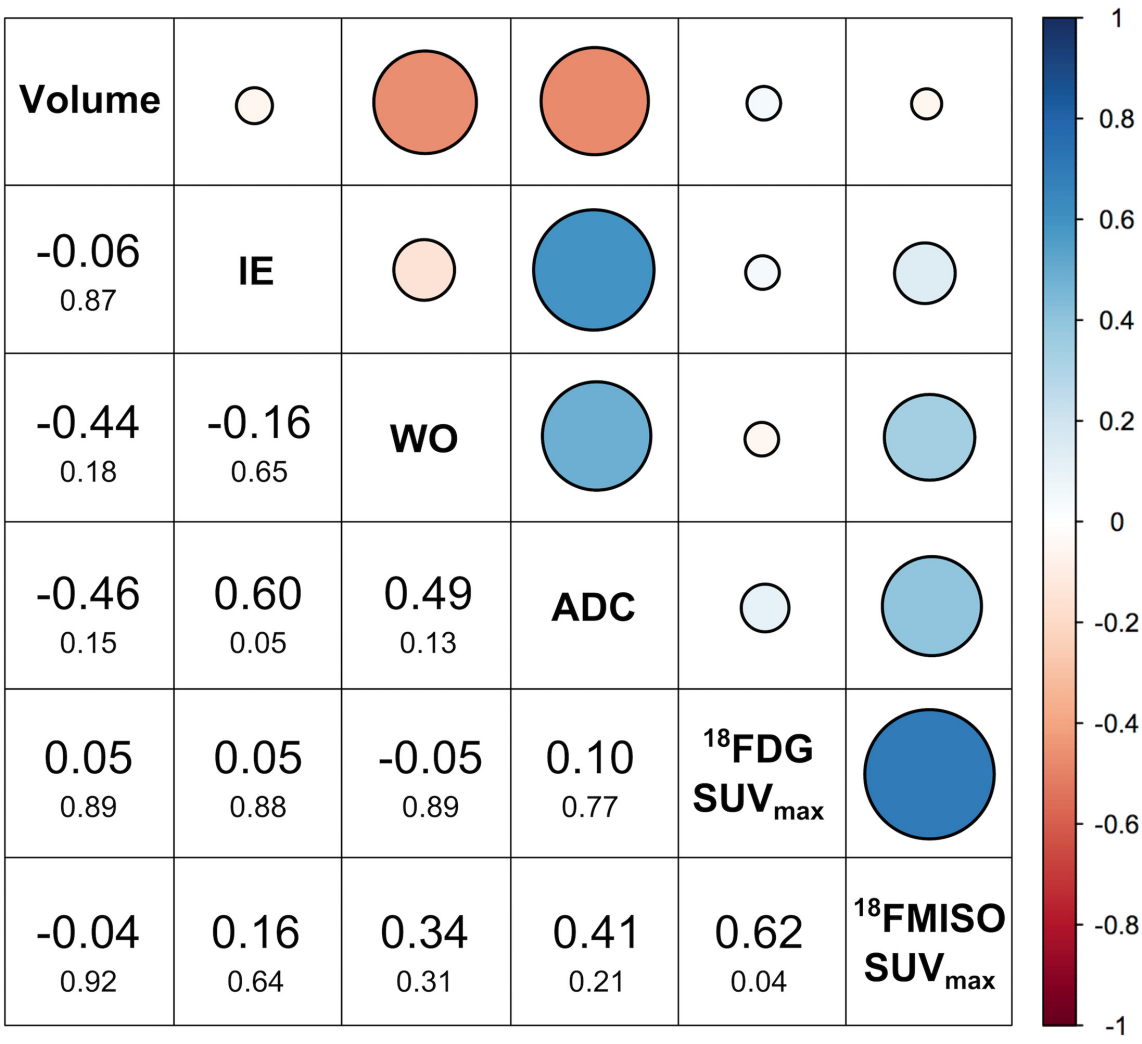
Summary of correlations between tumor volumes and MRI and PET parameters for all eleven patients using descriptive statistics. Blue indicates a direct and red an indirect (cf. legend on the right) correlation. Correlation coefficients are values dispalyed in larger font and the p-values in smaller font below.

Correlations between tumor volume, various MRI and PET parameters are summarized in Fig 3.

A statistically significant strong direct correlation was found for [^18^F]FDG SUV_max_ and[^18^F]FMISO SUV_max_ (p = 0.04) demonstrating that metabolically active tumors present with hypoxic areas. Furthermore a statistically significant strong direct correlation of initial enhancement with ADC (p = 0.05) was obseeved, which likely reflects microperfusion effects known to slightly increased ADC values in the tumor [40, 41]. There was moderate direct correlation of wash-out rate with ADC indicating the tumor presenting with a wash-out have lower ADC values. Extracellular space changes (e.g. by increased cellularity) affect both ADC and wash-out chrarcteristcs [42]. There was a moderate indirect correlation for tumor volume with wash-out rate and with ADC and no correlation of tumor volume with [^18^F]FDG SUV_max_ and with [^18^F]FMISO SUV_max_.

### Voxel-by-voxel analysis

**Fig 4:**
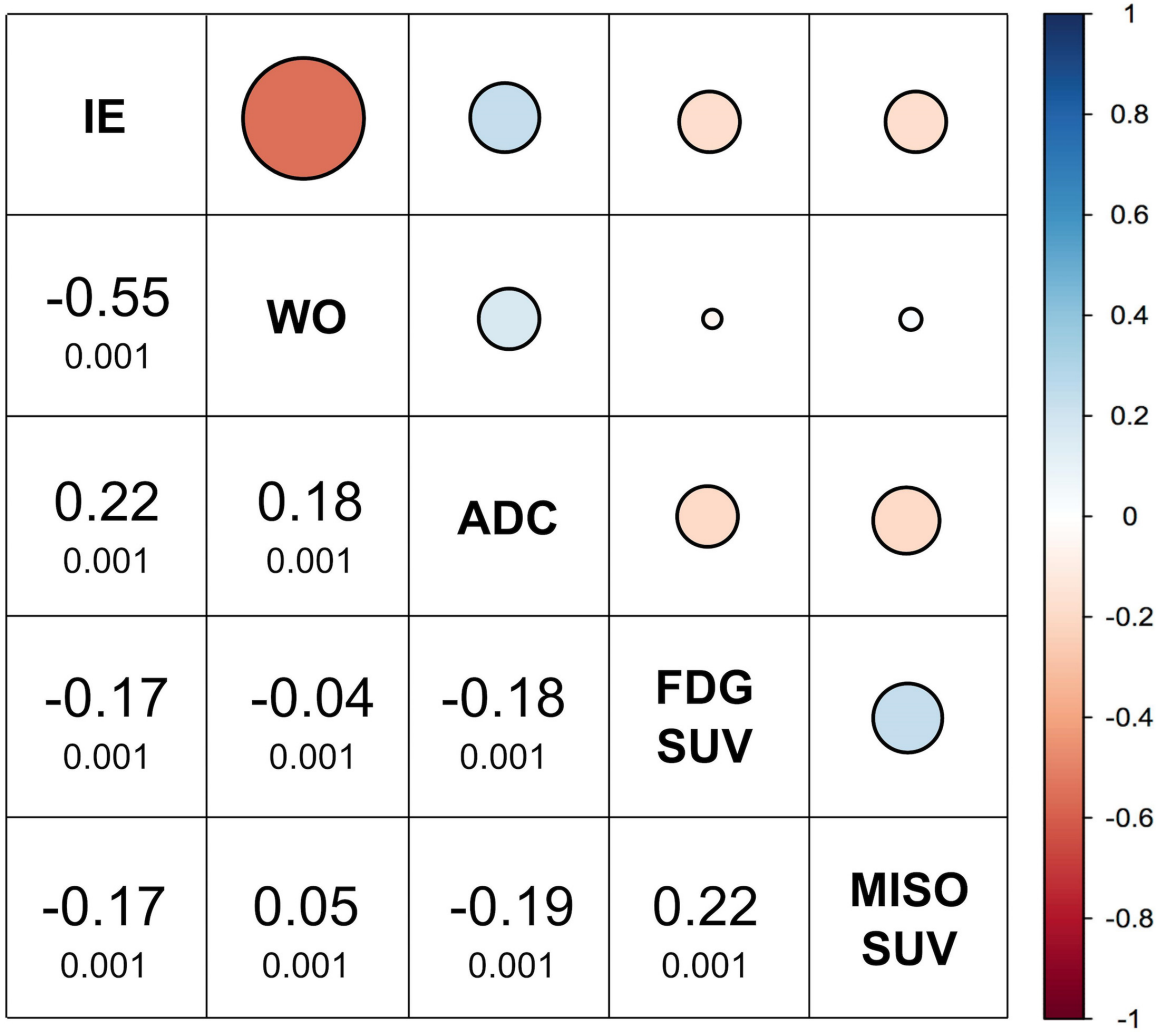
Correlations between tumor volumes, MRI, and PET parameters for eight patients using voxel-by-voxel analysis. Blue indicates a direct and red an indirect (cf. legend on the right) correlation. Correlation coefficients are values dispalyed in larger font and the p-values in smaller font below.

A voxel-by-voxel analysis of MP MRI and PET information was successfully performed in eight patients. In three patients DIR was not satisfactory due to either location of the tumor (n = 2) and too poor quality of the low-dose attenuation correction CT for [^18^F]FMISO. Fig 4 summarizes the respective correlations between MRI and PET parameters for these eight patients.

Except for initial and delayed enhancement, which showed a significant strong indirect correlation, i.e. tumors that show fast initial enhancement present with a wash-out, all other correlation of MRI and PET parameters were weak, ranging from 0.05–0.22. A weak direct correlation was found for [^18^F]FDG and [^18^F]FMISO SUV_max_.

## Discussion

MP [^18^F]FDG/[^18^F]FMISO PET/MRI facilitates the acquisition of a multitude of predictive and prognostic imaging parameters (CE-MRI, DWI, [^18^F]FDG, and [^18^F]FMISO PET), and each one yields independent information. MP [^18^F]FDG/ [^18^F]FMISO PET/MRI enables insights into tumor biology on multiple levels. This technique also provides information on tumor heterogeneity that in the future can be used for personalized cancer treatment by introducing dose-painting concepts with inhomogeneous radiation dose prescription and/or intensified chemotherapy regimens [2–6, 11].

Functional imaging with MP MRI is now part of the standard imaging protocol for treatment planning and prognostication for locally advanced cervical cancer [2–6, 11–14]. CE-MRI provides high-resolution anatomic information, depicts neo-angiogenesis, and quantifies the extent of poorly perfused regions within cervical tumors [3, 15, 17, 18, 43]. Poorly perfused tumor regions have been found to be an independent predictor of recurrence and survival [16–20, 44]. DWI provides information about tissue cellularity, which is an important factor in tumor response to CRT [22]. In addition, DWI has been identified as an imaging biomarker in cervical cancer for the early monitoring of response to CRT [22, 45]. Nevertheless, valuable information on metabolic and hypoxic tumor sub-volumes, which is pivotal for treatment planning, especially with regard to dose-painting with CRT, is limited with MRI alone.

[^18^F]FDG PET defines the extent of metabolically active disease, and thus, allows tailoring radiation treatment to the individual patient [5, 6], while avoiding irradiation of normal tissue. [^18^F]FDG PET facilitates the longitudinal tracking of metabolic disease during CRT, and the respective metabolic response to CRT is predictive of long-term survival [4, 5]. Tumor hypoxia is associated with increased resistance to ionizing radiation and various types of chemotherapy, thus diminishing the rate of local control as well as distant disease control [23–28]. Previous methods to measure and track tumor hypoxia comprised solely invasive assessment with needle electrodes or biopsy [23, 27, 28, 31]. However, in the past years different radiotracer such as [^18^F]FMISO, [^18^F]Fluoroazomycinarabinofuranoside [^18^F]AZA or [^64^Cu]-diacetyl-bis(N4-methylthiosemicarbazone) [^64^Cu] ATSM that can non-invasively identify such hypoxic tumor sub-volumes, which require radiation dose-escalative have been developed. In the current study we used the radiotracer [^18^F]FMISO as it is readily available in our institution and has been already clinically validated in RT for other tumors [46]. By combining the multitude of information from all the available different parameters, MP PET/MRI has the potential to provide accurate predictors of radio-curability for improved treatment planning and prognostication. Based on the results and feasibility of this pilot study, an on-going response assessment study was initiated, based on the same MP PET/MRI protocol. Respective imaging is performed pretreatment, during the 2^nd^ and 5^th^ week of CRT, as well as 3 months after treatment. Results will be reported in a separate communication.

As expected in this study all tumors displayed imaging parameters concordant with cancer, i.e. suspicious enhancement kinetics, decreased ADC values, and [^18^F]FDG- and [^18^F]FMISO-avidity. In all patients, focal hypoxic tumor subvolumes within the gross tumor volume were identified, which are associated with an increased radio-resistance and would require dose escalation for optimal CRT [24, 47–49].

In descriptive lesion-based statistical analysis there was also a significant strong direct correlation of [^18^F]FDG SUV_max_ with [^18^F]FMISO SUV_max_ (p = 0.04) and a moderate direct correlation of wash-out rate with ADC. The latter indicates that highly metabolically active tumors with a high cellular and microvascular density are also prone to present with hypoxic areas further hinting at tumor aggressiveness. However, there was no correlation of tumor volume with [^18^F]FDG SUV_max_ and with [^18^F]FMISO SUV_max_. Thus focal tumor hypoxia can be present in smaller tumors and is not just reserved for very big lesions having outgrown their oxygen supply. This implies that larger lesions are not necessarily the most aggressive ones with respect to imaging biomarkers such as enhancement kinetics, ADC, glycolytic activity and tumor hypoxia. Additionally, only a moderate indirect correlation for tumor volume with wash-out rate and with ADC was determined.

Whereas in the descriptive statistics a statistically significant strong positive association of [^18^F]FDG SUV_max_ with [1^8^F]FMISO SUV_max_ (p = 0.04) and initial enhancement with ADC (p = 0.05) was found, the voxel-by-voxel analysis revealed only weak correlations of these individual parameters except for a significant strong negative correlation between initial and delayed enhancement (p = 0.001). This might indicate that highly metabolically active and cellular tumors are more likely to have poorly perfused hypoxic areas. The weak direct correlation of [^18^F]FDG and [^18^F]FMISO SUV_max_ underlines that high glycolytic activity and tumor hypoxia is present in different tumor areas and that these imaging biomarkers provide complementary information. All these findings highlight that an aggressive imaging tumor phenotype, i.e. high cellullar and microvascular density, high glycolytic activity and focal tumor hypoxia can already be identified in smaller tumors.

In this study all tumors showed features of an aggressive tumor type i.e. high cellullar and microvascular density, high glycolytic activity and focal tumor hypoxia. which indicates that patients could benefit from an intensified tailored treatment. Each individual parameter reports on a different aspect of tumor biology and previous studies have shown benefits for each with regards to either treatment planning, response or prognostication. Therefore, we believe that each parameter is necessary to identify aggressive tumors and that the combined information of all parameters should be used for treatment planning and tracking of the spatio-longitudinal dynamics to facilitate personalized treatment.

This was a pilot study and initial results are encouraging. Especially with regard to the development of other targeted radiotracers and novel radiopharmaceuticals, MP [^18^F]FDG/[^18^F]FMISO PET/MRI has the potential to have a significant impact on cervical cancer treatment as a surrogate marker of CRT efficacy and drug activity at the tumor microenvironment level. Nevertheless, this study has some limitations. The patient collective is small. More patients agreed to participate than actually completed this study. Multimodality imaging studies are demanding for the patients, especially if performed on two scanners. This was the main reason why about 30% of the patient did not complete it. Performing such comprehensive imaging with simultaneous PET/MRI scanners, which are currently being installed world-wide, will certainly increase patient compliance [50]. Furthermore, simultaneous PET/MRI will overcome the other limitation of this study with respect to image post-processing for data correlation. Loosing valuable patient information like in this study, where for three patients a voxel-by-voxel analysis could not be performed, will also be avoided. Larger-scale studies, as well as consecutive examination during CRT, will be needed and are currently on-going to corroborate these results and to fully elucidate the predictive and prognostic potential of MP [^18^F]FDG/ [^18^F]FMISO PET/MRI.

In conclusion, MP [^18^F]FDG/ [^18^F]FMISO PET/MRI in patients with cervical cancer is feasible and provides unique complementary information on tumor biology and heterogeneity. In addition to several established predictive and prognostic imaging parameters, MP [^18^F]FDG/ [^18^F]FMISO PET/MRI can also identify hypoxic tumor sub-volumes, which are more resistant to radiotherapy and necessitate dose-escalation, which could further improve therapy planning and assessment of treatment response.

## Supporting Information

S1 Fig Illustration of the deformable registration outcome. Solid (green or yellow) line structure is the uterus and cervix as defined on the T1-weighted MRI, dashed line—as defined on the CT. (A) CT; (B) T2-weighted MRI; (C) color-coded representation of the calculated deformation field; (D) CT deformed to match the structure on the T1-weighted MRI.
(TIFF)

S2 Fig Example of MP [^18^F]FDG/[^18^F]FMISO PET/MRI in a 54-year-old patient with locally advanced cervical cancer scheduled for CRT using rigid image registration. (A) MP [^18^F]FDG/[^18^F]FMISO PET/MRI shows a highly [^18^F]FDG-avid tumor of the cervix (B) with focal areas of [^18^F]FMISO uptake indicative of tumor hypoxia distribution.
(TIFF)
