# Supplementary figures and images for: Multiparametric [18F]Fluorodeoxyglucose/ [18F]Fluoromisonidazole Positron Emission Tomography/ Magnetic Resonance Imaging of Locally Advanced Cervical Cancer for the Non-Invasive Detection of Tumor Heterogeneity: A Pilot Study
Source: PLoS One. 2016 May 11;11(5):e0155333. doi: 10.1371/journal.pone.0155333 (PMC4864307; doi:10.1371/journal.pone.0155333)

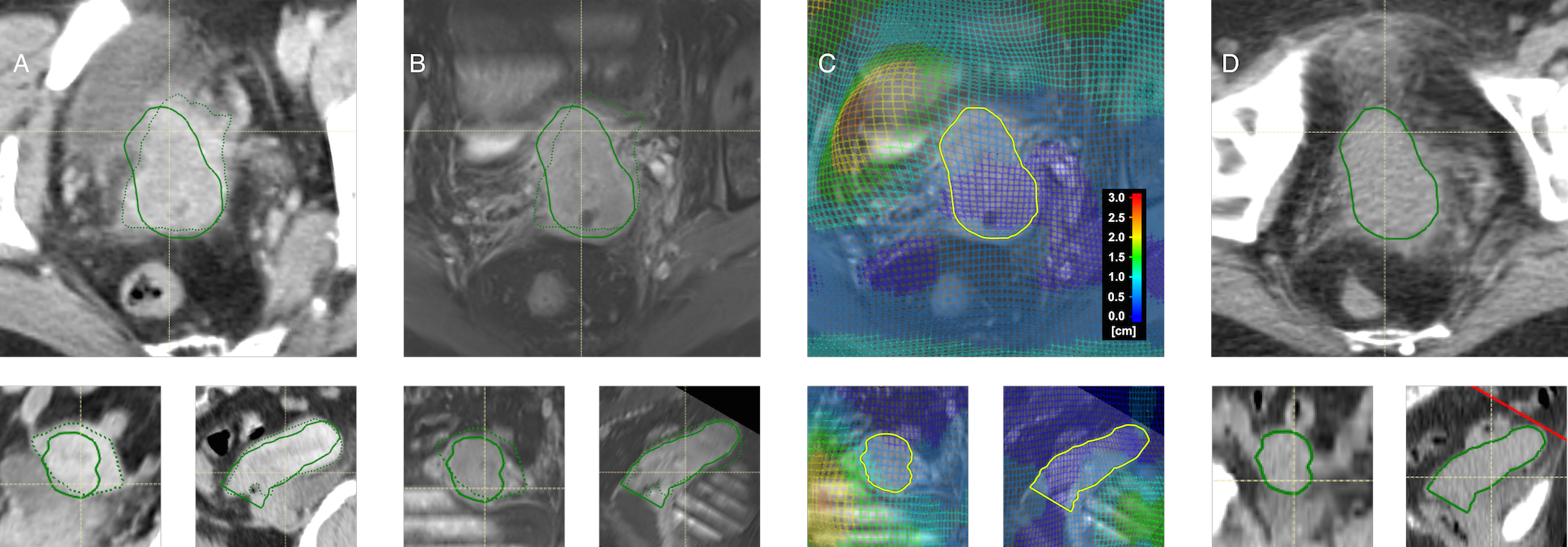

Supplement: S1 Fig — Solid (green or yellow) line structure is the uterus and cervix as defined on the T1-weighted MRI, dashed line—as defined on the CT. (A) CT; (B) T2-weighted MRI; (C) color-coded representation of the calculated deformation field; (D) CT deformed to match the structure on the T1-weighted MRI. (TIFF) [file pone.0155333.s001.tiff]

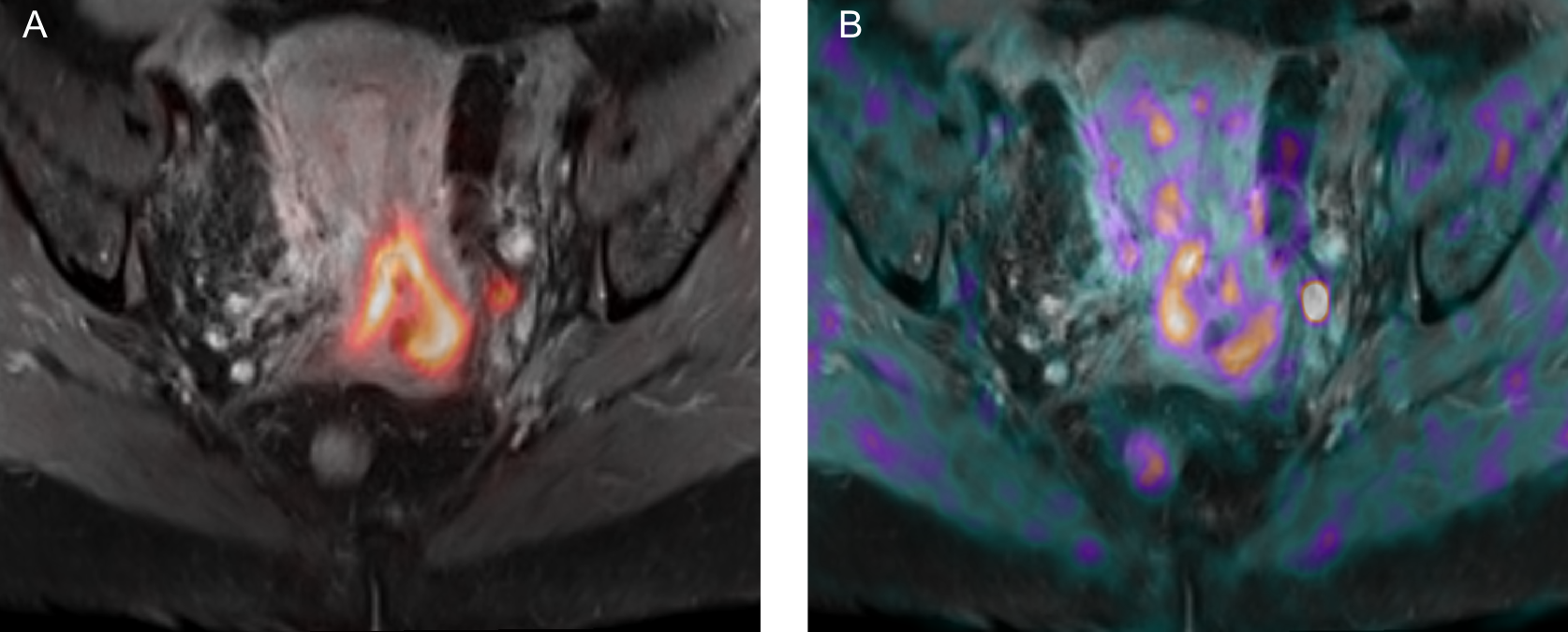

Supplement: S2 Fig — (A) MP [18F]FDG/[18F]FMISO PET/MRI shows a highly [18F]FDG-avid tumor of the cervix (B) with focal areas of [18F]FMISO uptake indicative of tumor hypoxia distribution. (TIFF) [file pone.0155333.s002.tiff]
